# Characteristics of rhizosphere and bulk soil microbial community of Chinese cabbage (*Brassica campestris*) grown in *Karst* area

**DOI:** 10.3389/fmicb.2023.1241436

**Published:** 2023-09-15

**Authors:** Xiaoliao Wei, Tianling Fu, Guandi He, Zhuoyan Zhong, Mingfang Yang, Fei Lou, Tengbing He

**Affiliations:** ^1^College of Agriculture, Guizhou University, Guiyang, China; ^2^Engineering Key Laboratory for Pollution Control and Resource Reuse Technology of Mountain Livestock Breeding, Institute of New Rural Development, Guizhou University, Guiyang, China

**Keywords:** microbial communities, enzymatic activity, soil properties, bacteria and fungi, co-occurrence network

## Abstract

Understanding the rhizosphere soil microbial community and its relationship with the bulk soil microbial community is critical for maintaining soil health and fertility and improving crop yields in *Karst* regions. The microbial communities in the rhizosphere and bulk soils of a Chinese cabbage (*Brassica campestris*) plantation in a *Karst* region, as well as their relationships with soil nutrients, were examined in this study using high-throughput sequencing technologies of 16S and ITS amplicons. The aim was to provide theoretical insights into the healthy cultivation of Chinese cabbage in a *Karst* area. The findings revealed that the rhizosphere soil showed higher contents of organic matter (OM), alkaline hydrolyzable nitrogen (AN), available phosphorus (AP), total phosphorus (TP), available potassium (AK), total potassium (TK), total nitrogen (TN), catalase (CA), urease (UR), sucrase (SU), and phosphatase (PHO), in comparison with bulk soil, while the pH value showed the opposite trend. The diversity of bacterial and fungal communities in the bulk soil was higher than that in the rhizosphere soil, and their compositions differed between the two types of soil. In the rhizosphere soil, Proteobacteria, Acidobacteriota, Actinobacteriota, and Bacteroidota were the dominant bacterial phyla, while Olpidiomycota, Ascomycota, Mortierellomycota, and Basidiomycota were the predominant fungal phyla. In contrast, the bulk soil was characterized by bacterial dominance of Proteobacteria, Acidobacteriota, Chloroflexi, and Actinobacteriota and fungal dominance of Ascomycota, Olpidiomycota, Mortierellomycota, and Basidiomycota. The fungal network was simpler than the bacterial network, and both networks exhibited less complexity in the rhizosphere soil compared with the bulk soil. Moreover, the rhizosphere soil harbored a higher proportion of beneficial Rhizobiales. The rhizosphere soil network was less complicated than the network in bulk soil by building a bacterial–fungal co-occurrence network. Furthermore, a network of relationships between soil properties and network keystone taxa revealed that the rhizosphere soil keystone taxa were more strongly correlated with soil properties than those in the bulk soil; despite its lower complexity, the rhizosphere soil contains a higher abundance of bacteria which are beneficial for cabbage growth compared with the bulk soil.

## Introduction

Rhizosphere microorganism has a vital function in fostering plant development and preserving plant health (Fan et al., 2017). The rhizosphere microbial diversity is greatly influenced by the bulk soil, and changes in the bulk soil microbial communities also impact the rhizosphere species diversity (Mendes et al., 2014). Edwards et al. (2015) proposed a multi-step model for the composition of soil microbes from roots in pure cultures or greenhouses, where each rhizosphere-associated compartment contains a distinct microbiome. In real agricultural systems, there is a decline in microbial diversity near the rhizosphere (Ling et al., 2022). However, most studies have focused solely on either fungal or bacterial communities (Zhang et al., 2017), and there has been limited research on the properties of microbial communities in the rhizosphere and bulk soils of crops and their response to rhizosphere effects (Fan et al., 2018). Microbial communities consist of species involved in symbiotic interactions that compete for food, energy substances, and spaces (Hibbing et al., 2010; Faust and Raes, 2012). Co-occurrence networks have been found particularly useful in studying complex relationships between numerous microbial species (Faust and Raes, 2012; Röttjers and Faust, 2018). These networks are widely used to research microbiome connections and provide valuable insights into microbial co-occurrence patterns and their critical mechanisms (Ma et al., 2020). Studies have shown that grape and soybean rhizosphere soil communities are subsets of their bulk soil communities in short-term farming systems, and the rhizosphere exhibited a simpler network of bacterial communities than bulk soil (Mendes et al., 2014). Fan et al. (2017) investigated the symbiotic patterns of three soils in the rhizosphere, such as bulk, loosely bound, and tightly bound rhizosphere in wheat and found distinct network structures and distribution patterns among them. Additionally, Wang et al. (2022) examined differences in compositional processes and bacterial community co-occurrence patterns in the rhizosphere and bulk soils with the addition of nitrogen, which were closely related to rhizosphere exudates. However, there is limited information available on topological changes in the Chinese cabbage rhizosphere bacterial and fungal symbiotic interactions in the *Karst* area compared with the bulk soil. In addition, keystone taxa play important roles in co-occurrence networks (Fan et al., 2018). Their removal may negatively impact microbiome stability and lead to significant changes in microbiome composition and function (Banerjee et al., 2018). Keystone taxa have been found in different environments (Fisher and Mehta, 2014; Fan et al., 2018), and they serve ecological functions in the rhizosphere, inhibiting fungal and bacterial pathogens (Mendes et al., 2011). Previous studies have identified a few keystone taxa and found that although they have an important role in the network, their relative abundance is relatively low (Shi et al., 2016). However, few studies have focused on the fungal and bacterial keystone taxa of *Karst* agroecosystems, let alone attempted to identify the soil properties that influence their distribution and interactions (Fan et al., 2018).

Chinese cabbage (*Brassica campestris*), a crucial cruciferous crop extensively cultivated and consumed in *Karst* areas (Nie et al., 2017), faces challenges due to the ecologically fragile nature of these landscapes (Wang et al., 2018), where agriculture plays a crucial role for livelihood (Chen et al., 2021). The cultivated land in this area features prominent exposure to carbonate rock, limited soil volume, shallow soil depth, and gradual soil development (Zhang et al., 2016), making land resources scarce. Therefore, improving crop productivity, especially Chinese cabbage, becomes crucial to meet the population's demand in this limited land (Timmusk et al., 2017). Particularly in this with restricted nutrient availability, the root system is essential to plant production (Camargo et al., 2023; Rüger et al., 2023). Previous studies have focused on the microbial community in the rhizosphere of Chinese cabbage to improve resistance and yield. For example, the biological control agent *Streptomyces alfalfae* XY25 T demonstrated promise in reducing clubroot disease, controlling rhizosphere bacterial and fungal populations in the rhizosphere of Chinese cabbage, and enhancing growth in greenhouse conditions (Hu et al., 2021); *Trichoderma harzianum LTR-2*, which works by modifying the rhizosphere soil microbial population, is discovered to be an efficient biological control agent for cabbage clubroot (Li et al., 2020). Additionally, three preceding crops were shown to increase Chinese cabbage yield and suppress clubroot disease by regulating the soil environment and microbial communities in the rhizosphere (Zhang et al., 2022). Furthermore, three arsenic-tolerant bacterial strains were isolated from the Chinese cabbage rhizosphere and bulk soils, and these strains were capable of promoting the growth of edible tissues while reducing the uptake of arsenic and cadmium by these tissues (Wang et al., 2017). Understanding microbial communities in the rhizosphere soil and their relationship with the microbial community in the bulk soil is essential for sustainable ecosystem functioning and improved crop yields in the *Karst* areas. However, there is limited research on the rhizosphere microorganisms of Chinese cabbage in *Karst* areas and their characterization in relation to the microbial community of the bulk soils and their relationship with soil nutrients. This study used high-throughput sequencing of 16S and ITS amplicons to investigate the bacterial and fungal populations in the rhizosphere and bulk soils of cabbage crops in *Karst* locations. The hypothesis was that the rhizosphere soils would exhibit a lower diversity of bacteria and fungi compared with bulk soils, and the community composition and co-occurrence networks would be simpler in the rhizosphere soils. Additionally, it was anticipated that the bacterial and fungal populations in the rhizosphere would be more impacted by the properties of the soil. The goals of this study are as follows: (1) to assess the differences in microbial community structure, potential functions, and diversity between rhizosphere and bulk soils of Chinese cabbage plantations in *Karst* regions; (2) to identify bacterial and fungal keystone taxa in rhizosphere and bulk soils of Chinese cabbage plantations in *Karst* regions. These findings will provide valuable theoretical data for promoting healthy cabbage cultivation in the *Karst* area.

## Materials and methods

### Study area and soil sampling

The study area covers ~0.35 square kilometers and is situated in Shiliping Town, Xingyi City, Guizhou Province, Southwest China's *Karst* area (24°58′ 0′′ N-25°1′ 0^′′^ N, 104°50^′′^ 0′ E-104°55′ 0^′′^ E) (Supplementary Figure S1). The plots measured 5 m × 5 m, with six replicates being sampled. In each plot, 25 Chinese cabbage plants were collected by the five-point sampling method. Loosely bound soil was shaken off, and tightly adhered soil was brushed off with sterilized brushes to collect the rhizosphere soil (*n* = 6). In addition to each group of Chinese cabbage plants, topsoil (0–20 cm) without plants was collected using a soil auger to serve as the bulk soil (*n* = 6). Approximately 50 g of soil was taken and placed in a sterile bag and stored in an icebox (4–10°C) in the field, and another portion was brought back to the laboratory in a sterile bag. The first part (50 g) of soil was stored in an −80°C ultra-low temperature refrigerator and used for soil microbial analysis, while the other part of the soil was placed in the room to air dry and then used for analysis of soil properties after grinding.

### Analyses of soil properties

Standard soil testing techniques were employed to assess various physical and chemical properties of soil, including organic matter (OM), alkaline hydrolyzable nitrogen (AN), total nitrogen (TN), available potassium (AK), total potassium (TK), available phosphorus (AP), and total phosphorus (TP) (Bao, 2000); soil pH was determined potentiometrically with water: soil ratio of 2.5:1 (v/w). Additionally, soil enzyme activity, namely catalase (CA), urease (UR), sucrase (SU), and phosphatase (PHO) in soil; potassium permanganate titration was performed to determine soil catalase activity (CA), expressed in milliliters of 0.1 N potassium permanganate in 1 g of soil after 20 min of shaking in a shaker. The sodium phenolate colorimetric method was used to determine urease (UR). The activity of urease was expressed as the milligrams of NH_3_-N in 1 g of soil present after incubation for 24 h in an incubator at 37°C. The sucrase (SU) was measured by the 3,5-dinitrosalicylic acid colorimetric method, and the activity of sucrase was expressed as the milligrams of glucose produced in 1 g of soil incubated after 24 h in an incubator at 37°C. The phosphatase (PHO) was measured by the disodium phenyl phosphate colorimetric method; after incubating in an incubator at 37°C for 24 h, the phenol mass (mg) released in 1 g of soil represented the activity of phosphatase (Zhou and Zhang, 1980).

### Extraction of soil DNA and sequencing of amplicon

Soil genomic DNA was extracted from 0.5 g of frozen soil (−80°C) utilizing the FastDNA^®^ Spin Kit for soil (MP Biomedicals, USA), following the manufacturer's protocol. The quality and concentration of DNA were determined by 1.0% agarose gel electrophoresis and a spectrophotometer of NanoDrop2000 (Thermo Scientific Inc., USA) and stored at −80°C before further use. The V3-V4 region of the bacterial 16S rRNA gene was amplified utilizing the 338F (5′-ACTCCTACGGGAGGCAGCAG-3′) and 806R (5′-GGACTACHVGGGTWTCTAAT-3′) primers (Liu et al., 2016), and the ITS1 region of ITS rRNA gene was targeted using ITS1F (5′-CTTGGTCATTTAGAGGAAGTAA-3′) and ITS2R (5′-GCTGCGTTCTTCATCGATGC-3′) primers (White et al., 1990; Gardes and Bruns, 1993). Each sample was subjected to triplicate amplification, and detailed steps are provided in the Supplementary Files. The PCR products were combined in equal proportions and sequenced on the Illumina MiSeq PE300 platform (Illumina, USA), following the standard protocol at Majorbio Bio-Pharm Technology Co., Ltd (Shanghai, China). The raw sequencing data were uploaded to the NCBI Sequence Read Archive database with the accession number PRJNA865816.

### Bioinformatics and statistics

The raw FASTQ files were processed utilizing QIIME to demultiplex and quality-filter them based on specific conditions (Caporaso et al., 2010), and detailed steps are provided in the Supplementary Files. The operational taxonomic units (OTUs) for bacteria and fungi were clustered at a 97% sequence similarity threshold, using the UPARSE program, and chimeric sequences were removed using UCHIME (Edgar et al., 2011; Edgar, 2013). To keep the number of valid sequences consistent for each sample, the original sequence was drawn flat according to the minimum sample sequence. OTUs not belonging to soil bacterial and fungal communities were excluded, resulting in 6,862 bacterial and 1,501 fungal OTUs. The taxonomic composition of bacterial and fungal OTUs was determined using the Bayesian-based RDP classifier method with data from SILVA and UNITE databases. Functional prediction analysis of the 16S rRNA gene and ITS rRNA gene was performed utilizing FAPROTAX and FUNGuild on the Majorbio Cloud Platform. Correlation analysis and ANOVA were conducted utilizing SPSS statistical software (IBM Corporation, USA). The relative abundance of microorganisms is the proportion of specific bacteria or fungi among all bacteria or fungi, expressed as a percentage. The ggplot2, ggvenn, reshape2, d3Network, and circlize packages of R were used to create clustered heatmaps, boxplots, Venn diagrams, stacked percentage plots, Sankey diagram, and chord diagram, respectively (Wei et al., 2023). Moreover, a principal coordinate analysis (PCoA) was performed utilizing the R vegan package, and PERMANOVA, with 999 permutations, was utilized to validate the PCoA findings according to the Bray–Curtis distance. The Mantel test was achieved utilizing the vegan package in R software (Yang et al., 2017). RDA analysis was chosen based on the gradient lengths of the first axis in the DCA analysis results for bacteria and fungi (1.10 and 2.08, respectively), and its visualization was referenced from previous research (Wei et al., 2023). Network analysis was conducted according to Spearman (Spearman's *r* <- 0.7 or *r* > 0.7; *P* < 0.05), and other screening conditions and visualizations were followed from previous studies (Wei et al., 2023). The igraph tool in R was used to create the co-occurrence network, and Gephi was utilized for visualization (Zhang et al., 2018a). According to descriptions from earlier studies, keystone taxa were chosen (Liu et al., 2022).

## Results

### Analysis of soil properties and microbial diversity

**Figure 1:**
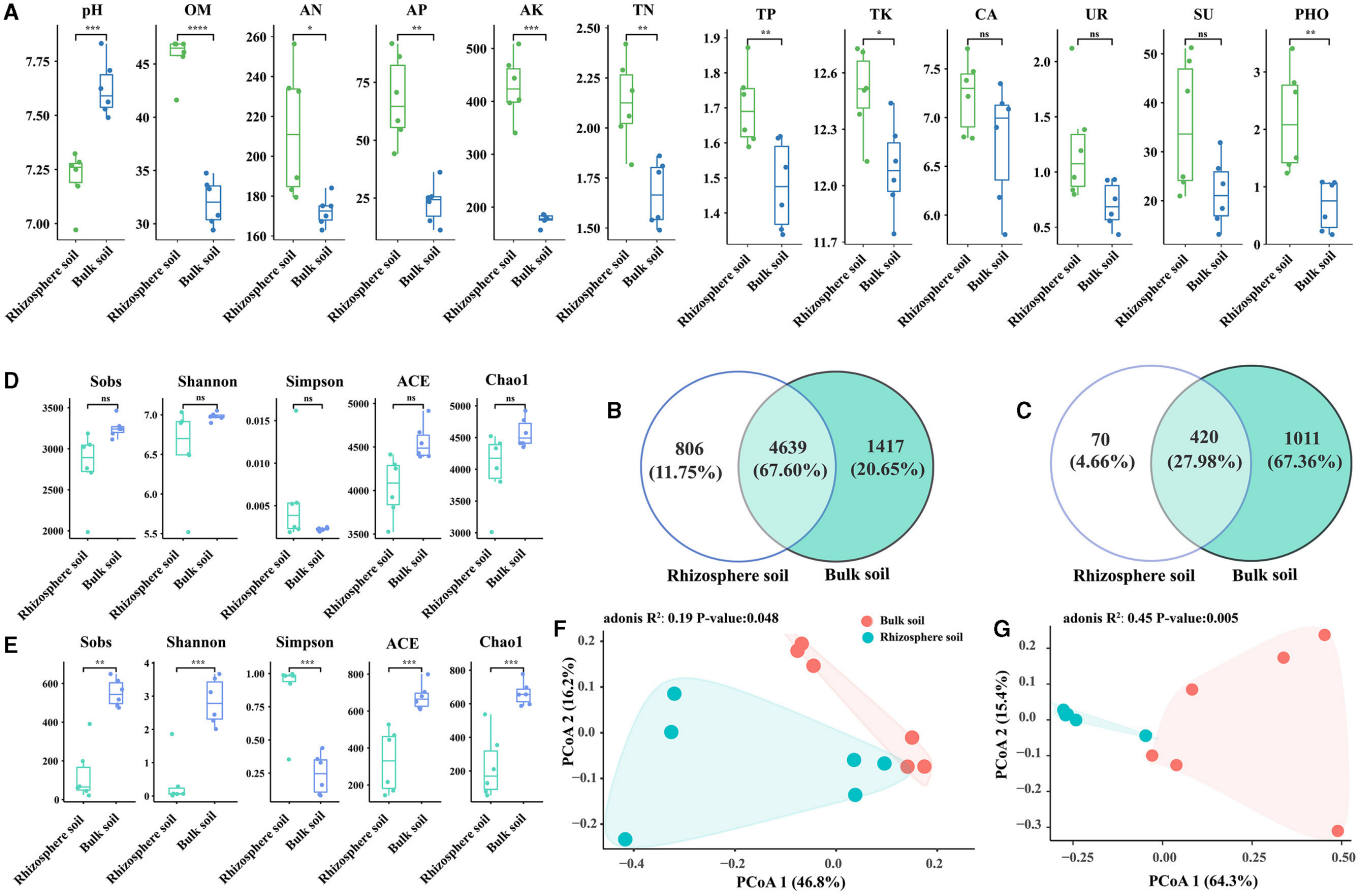
Variations in soil properties **(A)**, OTU numbers **(B, C)**, and diversity **(D–G)** between the rhizosphere soil and bulk soil of Chinese cabbage; *t*-tests were conducted for soil properties **(A)**; the number of bacterial OTUs **(B)**; the number of fungal OTUs **(C)**; bacterial alpha diversity **(D)**; fungal alpha diversity **(E)**; PCoA analysis of bacterial beta diversity **(F)**; and fungal beta diversity **(G)**. The indicators such as pH, OM, AN, AP, AK, TN, TP, TK, CA, UR, SU, and PHO represent hydrogen ion concentration, organic matter, alkali hydrolyzable nitrogen, available phosphorus, available kalium, total nitrogen, total phosphorus, total potassium, catalase, urease, sucrase, and phosphatase, respectively. “ns” shows at *p* > 0.05, “*” shows at *p* ≤ 0.05, “**” shows at *p* ≤ 0.01, “***” shows at *p* ≤ 0.001, and “****” shows at *p* ≤ 0.0001.

In comparison with bulk soils, the rhizosphere of Chinese cabbage showed higher contents of organic matter (OM), alkaline hydrolyzable nitrogen (AN), available phosphorus (AP), total phosphorus (TP), available potassium (AK), total potassium (TK), total nitrogen (TN), catalase (CA), urease (UR), sucrase (SU), and phosphatase (PHO), while the pH value showed the opposite trend. These differences were statistically significant, except for CA, UR, and SU (Figure 1A). Bacterial species were more abundant than fungal species, and the rhizosphere soil had a lower number of OTUs than the bulk soil (Figures 1B, C). Notably, the rhizosphere soil exhibited lower bacterial and fungal alpha diversity values for indices such as Sobs, Shannon, ACE, and Chao1 compared with the bulk soil. Nevertheless, the Simpson index exhibited the contrary pattern, suggesting that the rhizosphere soil had a lower alpha diversity of bacteria and fungi than bulk soil. Additionally, there was a significant difference in the alpha diversity of fungi between rhizosphere and bulk soils (*p* < 0.05), while bacterial alpha diversity did not exhibit significant variation (*p* > 0.05) (Figures 1D, E). The correlation analysis showed a positive relationship between bacterial Shannon index and fungal Shannon index, and a similar pattern was observed for other indices (except for Shannon and Simpson indices) (Supplementary Table S1). Furthermore, the PCoA results for communities of bacteria and fungi displayed clear differences between bulk and rhizosphere soils (Figures 1F, G).

### Composition of microbial community

**Figure 2:**
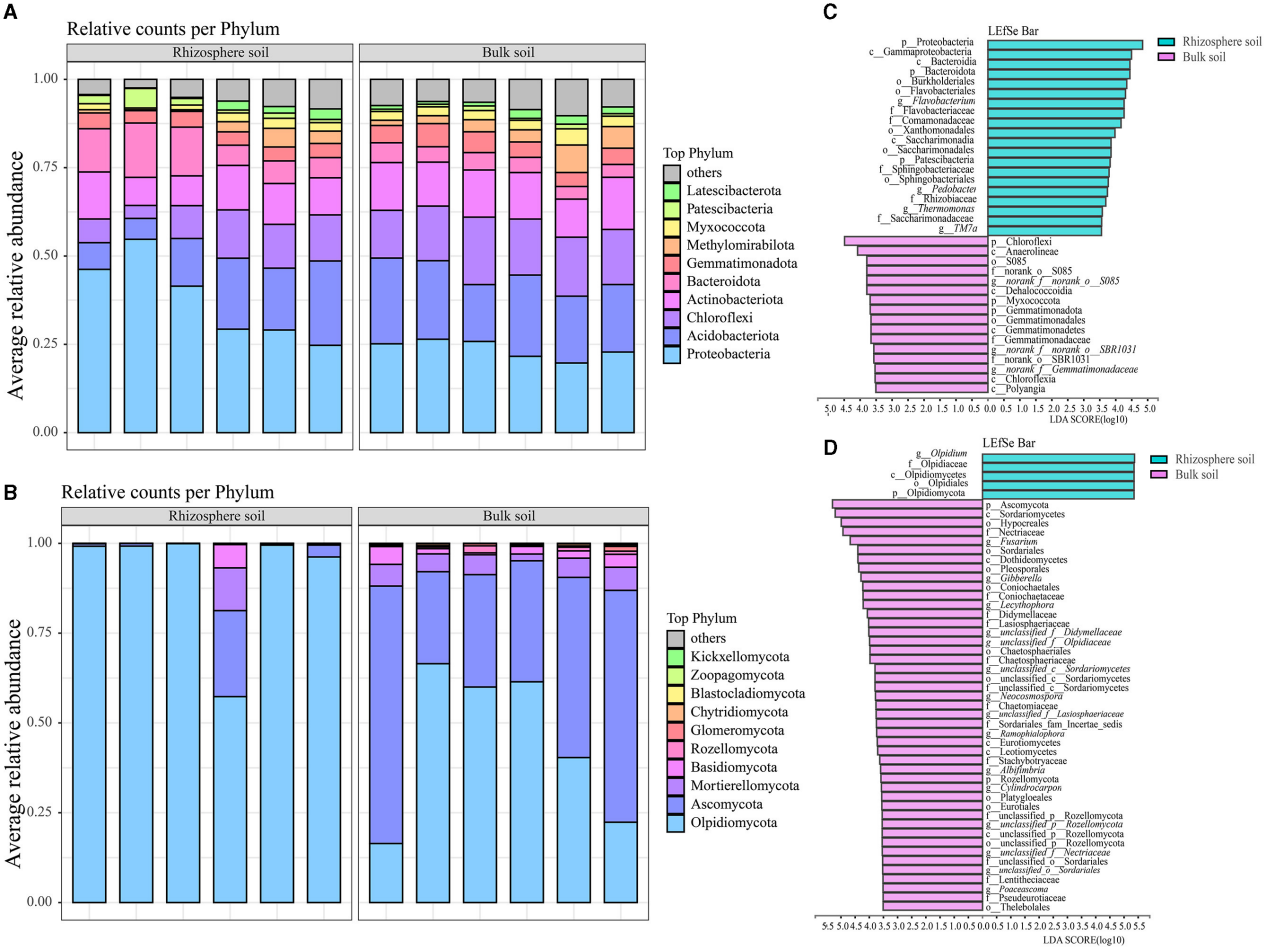
Composition and LefSe analysis of bacterial and fungal communities in both the rhizosphere soil and bulk soil of Chinese cabbage. **(A, B)** represent soil bacterial and fungal composition (at the phylum level), respectively; **(C, D)** stand for LefSe analysis results for bacteria and fungi (respectively, ranging from the phylum to genus level).

Among the 42 bacterial and 10 fungal phyla identified, 4 major phyla collectively accounted for over 70% of the total relative abundances in both rhizosphere and bulk soils of the *Karst* region, where Chinese cabbage was cultivated (Figures 2A, B and Supplementary Table S2). Proteobacteria, Acidobacteriota, Actinobacteriota, and Bacteroidota were dominant bacterial phyla in the rhizosphere soil, representing 37.59, 14.74, 10.73, and 9.87% of the total relative abundances, respectively. In contrast, Proteobacteria, Acidobacteriota, Chloroflexi, and Actinobacteriota were dominant bacterial phyla in the bulk soil, accounting for 23.60, 20.60, 16.03, and 13.00% of the total relative abundances, respectively (Figure 2A and Supplementary Table S2). The rhizosphere soil had higher relative abundances of *Olpidiomycota, Ascomycota, Mortierellomycota*, and *Basidiomycota*, which accounted for 91.89, 4.86, 2.03, and 2.03% of the total relative abundances, respectively. In comparison, the bulk soil had higher relative abundances of *Ascomycota, Olpidiomycota, Mortierellomycota*, and *Basidiomycota*, accounting for 46.16, 44.52, 5.03, and 2.44% of the total relative abundances, respectively (Figure 2B and Supplementary Table S2). Notably, there were significant differences in the fungal phyla between the bulk and rhizosphere soils. Particularly, the relative abundance of *Olpidiomycota* in the rhizosphere (91.89%) was approximately double the amount in the bulk soil (44.52%), whereas the relative abundance of *Ascomycota* was ~10 times higher in the bulk soil (46.16%) than in the rhizosphere soil (4.86%). These findings indicate notable disparities in bacterial and fungal communities between the Chinese cabbage rhizosphere and bulk soils. LefSe analysis (LDA > 3.5) revealed remarkable variations at the taxonomy levels from phylum to genus between the bulk soils and rhizospheres (Figures 2C, D). Specifically, a greater number of bacterial taxa, including *Proteobacteria, Gammaproteobacteria*, and *Bacteroidia*, were enriched in the rhizosphere of Chinese cabbage compared with the bulk soils. In addition, the rhizospheres exhibited higher relative abundances of fungi such as *Olpidium*, Olpidiaceae, and *Olpidiomycetes*, compared with the bulk soils (Figure 2C). In contrast, a higher number of fungal taxa, such as *Chloroflexi, Anaerolineae*, and *S085*, were enriched in the Chinese cabbage bulk soils, accompanied by higher fungal relative abundances such as Ascomycota, Sordariomycetes, and Hypocreales, compared with the rhizosphere soils (Figure 2D).

### Differences in the distribution of bacteria and fungi

**Figure 3:**
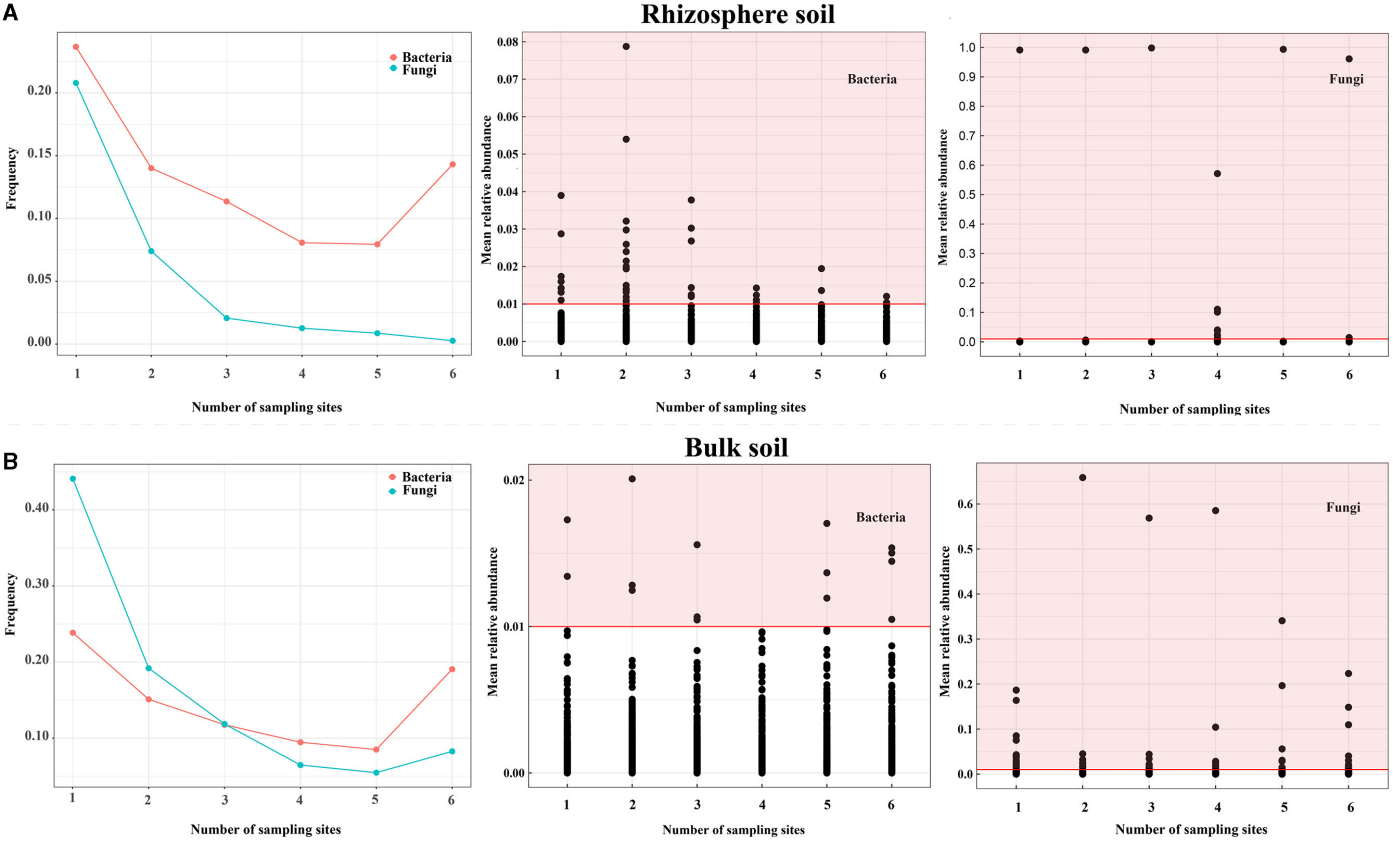
Distribution of soil bacteria and fungi of Chinese cabbage in the *Karst* area. **(A, B)** show the distribution frequency of bacterial and fungal OTUs, the OTUs that bacteria have a relative abundance of >1%, and the OTUs that fungi have a relative abundance of >1% in the rhizosphere and bulk soils, respectively. Notes: The red highlighted area indicates the number of bacterial and fungal OTUs with a relative abundance of > 1% in all sampling points for both the rhizosphere and bulk soils.

To assess the dissimilarities in the distribution of bacterial and fungal communities between bulk and rhizosphere soil (soils at the OTU level), the OTU distributions at each sampling point were analyzed (Figure 3A and Supplementary Table S3). Bacteria were more abundant than fungi across all sites (Figure 3A and Supplementary Table S3), while fungi exhibited more OTUs with a relative abundance of >1% at all sites (0.16% for fungi versus 0.1% for bacteria) in the rhizosphere soil (Figure 3A). However, most fungi were present at ≤3 sampling point OTU (>75% of OTUs), whereas bacteria were more prevalent at >3 sites (Figure 3B and Supplementary Table S3) in the bulk soil. Moreover, fungi exhibited more OTUs with a relative abundance of >1% in all sites (0.71% for fungi vs. 0.04% for bacteria) in the bulk soil (Figure 3B). To identify core OTUs, criteria were applied for OTUs that appeared in all samples with an average relative abundance of >0.1% in each group. In the rhizosphere soil, core OTUs comprised all OTUs (0.20% for fungi and 2.67% for bacteria) and were mainly distributed across multiple bacterial and fungal phyla, such as Proteobacteria, Acidobacteriota, Bacteroidota, Olpidiomycetes, and Ascomycota. In the bulk soil, core OTUs represented all OTUs (2.89% for bacteria and 3.46% for fungi) and were typically distributed across multiple bacterial and fungal phyla, such as Acidobacteriota, Actinobacteriota, Chloroflexi, Ascomycota, and Mortierellomycota (Supplementary Table S4). These findings indicated that bacterial and fungal communities in rhizosphere and bulk soils exhibit distinct distribution patterns, and bacteria generally have a wider distribution range compared with fungi.

### Correlation between soil properties and microbial community

**Figure 4:**
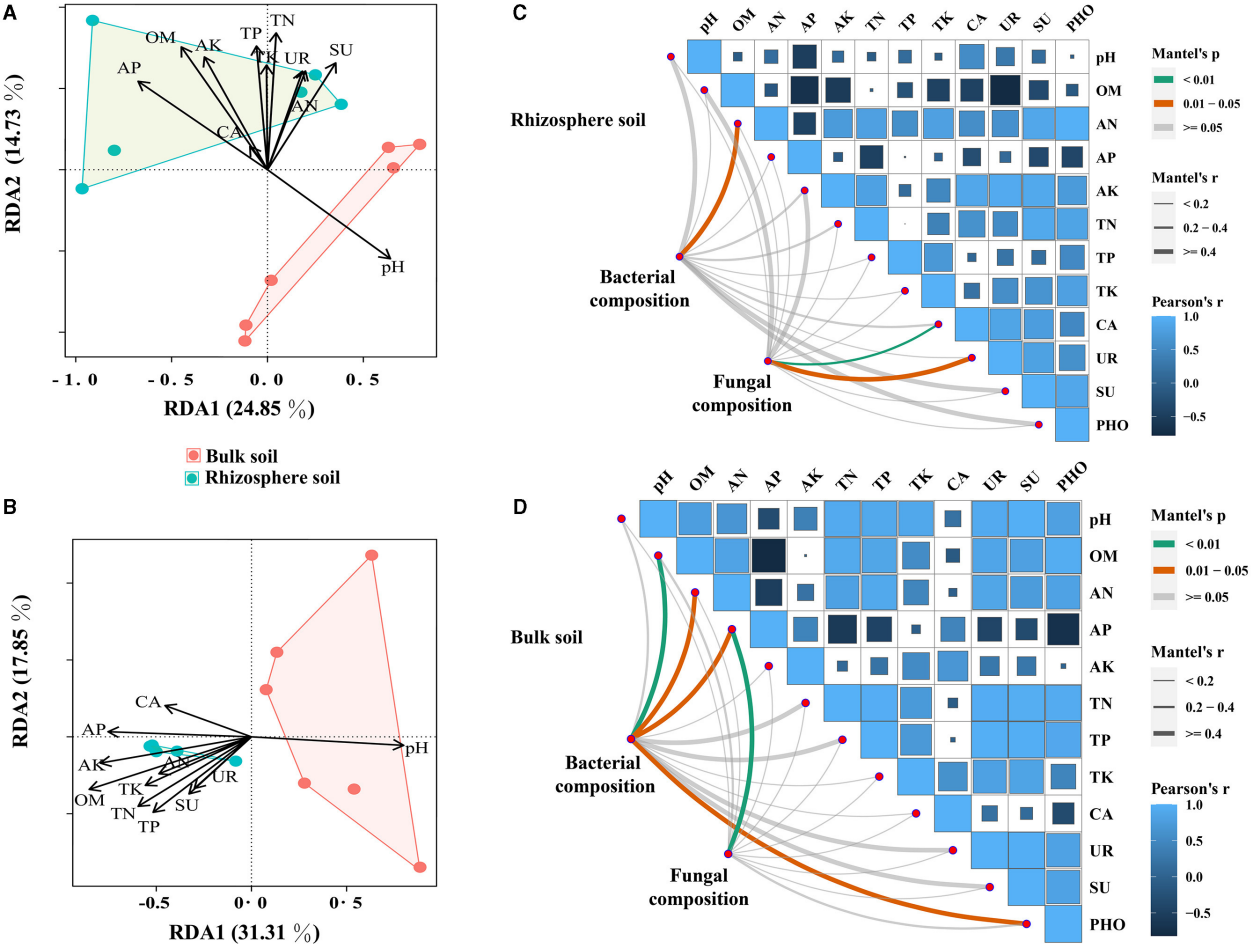
Mantel's test and RDA analysis between soil properties and bacterial and fungal communities (at the genus level). **(A, B)** represent the Mantel test results for the correlation between soil bacterial and fungal communities and soil properties, respectively. **(C, D)** represent RDA analysis results for the correlation between soil bacterial and fungal communities and soil properties, respectively.

Herein, the impact of soil properties on bacterial and fungal communities in relative abundance was investigated. To achieve this, Pearson's correlation analysis on the top 20 genera in relative abundance was conducted to assess their correlation with soil properties. The results revealed significant associations between several bacterial and fungal taxa and soil properties, with pH, AP, OM, and AK showing particularly strong correlations (Supplementary Figure S2). Notably, a positive correlation between pH and the dominant bacterial and fungal genera was found, while other soil properties exhibited opposite trends (Supplementary Figure S2 and Supplementary Table S5). Furthermore, the results of RDA for communities of bacteria and fungi demonstrated that OM had the most pronounced impact, displaying the highest correlation coefficients (Figures 4A, B and Supplementary Table S6). The Mantel test results suggested that soil properties had a greater impact on the microbial community in bulk soil compared with rhizosphere soils (Figures 4C, D and Supplementary Table S7). Additionally, the associations between soil properties were explored, and it was found that OM had significant positive correlations with AN, AN, AP, AK, TN, TP, and TK (Supplementary Table S8).

### Bacterial and fungal functional analyses

**Figure 5:**
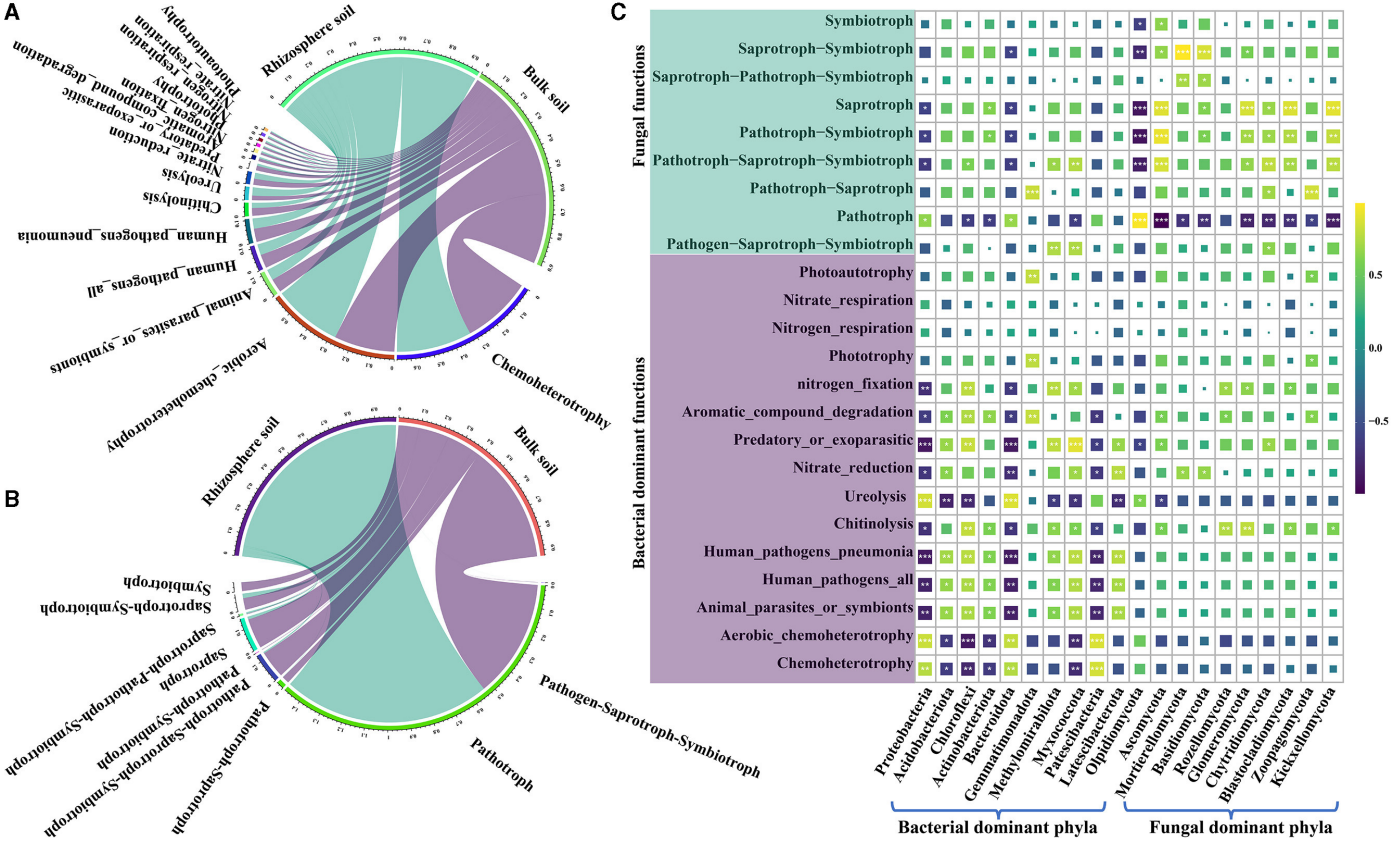
Functional prediction of bacterial FAPROTAX and fungal FUNGuild along with their correlation analysis with the dominant phyla. **(A)** Functional groups of the top 15 bacteria in the relative abundance. **(B)** functional groups of the top 15 fungi in the relative abundance. **(C)** correlation analysis between the dominant functional groups of soil bacteria and fungi and dominant bacterial phyla. “*” shows at 0.01 < *p* ≤ 0.05, “**” shows at 0.001 < *p* ≤ 0.01, and “***” shows at *p* ≤ 0.001.

The functional groups of bacterial communities in both rhizosphere and bulk soils of a *Karst* region were analyzed using FAPROTAX. In the rhizosphere soil, 49 functional groups were identified; in the bulk soil, 47 functional groups were found (Figure 5A and Supplementary Table S9). The major bacterial functional groups included chemoheterotrophy, aerobic chemoheterotrophy, animal parasites or symbionts, human pathogen-causing pneumonia, chitinolysis, ureolysis, nitrate reduction, predatory or ectoparasitic, and aromatic compound degradation. Notably, bacterial communities in the rhizosphere soil exhibited higher levels of chemoheterotrophic and aerobic chemoheterotrophic activities and showed superior ureolysis abilities compared with the bacteria in the bulk soil (Supplementary Table S9). On the other hand, the bulk soil displayed a higher bacterial functional group in relative abundance associated with predatory or ectoparasitic activity and aromatic compound degradation compared with the rhizosphere soil. Moreover, bacterial pathogens were found to be less prevalent in the rhizosphere soil than in the bulk soil (Figure 5A and Supplementary Table S9). For the fungal communities, FUNGuild analysis was employed to classify their functional groups. It was found that 30.91% of the fungal OTUs were not classified into functional groups present in the FUNGuild database. Nevertheless, fungal OTUs with a confidence level of at least “probable” for analysis account for 92.43 to 99.25% of fungi in the total relative abundance (Figure 5B, Supplementary Table S9). The most prevalent trophic modes of fungi were pathotroph, saprotroph–symbiotroph, saprotroph, pathotroph–symbiotroph, and pathotroph–saprotroph–symbiotroph. Among these, pathotroph, saprotroph–symbiotroph, and saprotroph were more abundant in the rhizosphere soil, constituting 94.42, 2.04, and 1.29% of the total relative abundance, respectively. In the bulk soil, the dominant trophic modes were mainly pathotroph, saprotroph, and pathotroph–saprotroph–symbiotroph, accounting for 53.56, 13.56, and 12.41% of the total relative abundance, respectively (Supplementary Table S9). Surprisingly, the relative abundance of pathotrophs in the rhizosphere was approximately twice as high as that in the bulk soil, while the relative abundance of saprotrophs was 10 times higher in the bulk soil than in the rhizosphere (Supplementary Table S9). Furthermore, Pearson's correlation analysis was conducted to research the association between functional groups and the most dominant bacterial and fungal phyla. The results showed significant correlations between the majority of bacterial and fungal functional groups and their respective dominant phyla (Figure 5C and Supplementary Table S10). Additionally, certain functional groups of bacteria exhibited significant correlations with dominant fungal phyla, while some fungal functional groups correlated with dominant bacterial phyla (Figure 5C and Supplementary Table S10). Notably, a strong positive correlation between Ascomycota and saprotroph and a significant negative correlation between Olpidiomycota and saprotroph were found (Supplementary Table S10). These findings indicate meaningful associations between dominant phyla and functional groups in the soil microbial communities.

### Analysis of co-occurrence network

**Figure 6:**
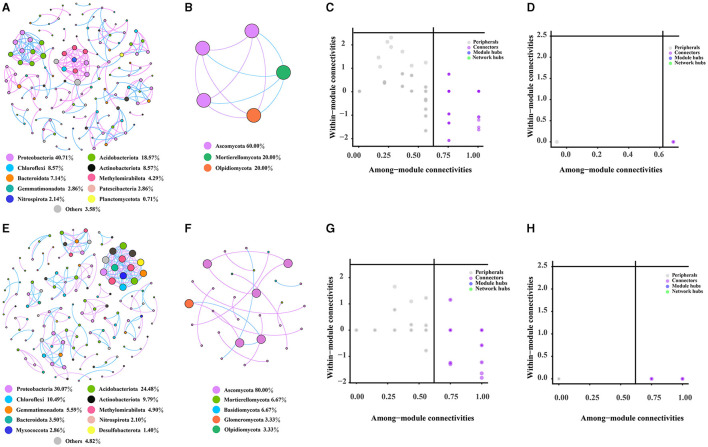
Characteristics of the rhizosphere and bulk soil networks. **(A, B)** represent the co-occurrence network of bacteria and fungi in the rhizosphere soil, respectively. **(C, D)** represent the keystone taxa of rhizosphere soil bacterial and fungal co-occurrence network, while **(E, F)**, respectively, represent the co-occurrence network of bacteria and fungi in the bulk soil. **(G, H)** show the keystone taxa of the bacterial and fungal co-occurrence network in the bulk soil.

The bacterial, fungal, and bacterial–fungal co-occurrence networks in the rhizosphere and bulk soils of Chinese cabbage in a *Karst* region were investigated. Comparing the fungal networks, it was observed that bacterial networks had a greater number of nodes and edges in the rhizosphere and bulk soils (Figures 6A, E). The bulk soils were predicted to harbor a higher number of keystone OTUs belonging to bacterial and fungal categories, specifically Burkholderiales, Vicinamibacterales, Rhizobiales, and Hypocreales. Notably, Burkholderiales, Vicinamibacterales, and Rhizobiales were found to exhibit higher levels of enrichment in the rhizosphere compared with the bulk soils. The bacterial network showed a higher degree of complexity than the fungal network, as indicated by network characteristics such as nodes, edges, and keystone taxa (Supplementary Table S11). However, both networks in the rhizosphere soil were found to be less complex compared with those present in the bulk soils (Figures 6A–H). Additionally, it was observed that the proportion of bacteria (97.24%) far exceeded that of fungi (2.76%) (Supplementary Figure S3A); the majority of nodes belonged to *Proteobacteria* (39.31%), *Acidobacteriota* (17.93%), *Chloroflexi* (8.28%), and *Actinobacteriota* (8.28%) in the bacterial–fungal network in the rhizosphere soil. In the bulk soil, the bacterial–fungal network dominated most nodes, including *Proteobacteria* (23.35%), *Acidobacteriota* (19.29%), *Ascomycota* (17.77%), and *Chloroflexi* (8.12%), while the proportion of bacteria (77.16%) was considerably higher than that of fungi (22.84%) (Supplementary Figures S3B, D). Furthermore, based on various network topological characteristics, the analysis revealed that the rhizosphere soil bacterial–fungal network (with 145 nodes, 249 edges, and 35 modules) exhibited lower complexity compared with the bulk soil network (such as 197 nodes, 370 edges, and 49 modules) (Supplementary Figures S4A–C). Keystone taxa that have a significant role in the network structure of both rhizosphere and bulk soil bacterial–fungal networks are identified. Specifically, 73 OTUs were identified as keystone taxa in the rhizosphere soil network, with the majority belonging to Burkholderiales, Vicinamibacterales, and Hypocreales orders (Supplementary Figure S4E and Supplementary Table S11). In the bulk soil network, 86 OTUs were identified as keystone taxa, with the majority belonging to the Vicinamibacterales, Hypocreales, and Burkholderiales orders (Supplementary Figure S4F and Supplementary Table S12). Notably, the rhizosphere soil had a higher abundance of Burkholderiales compared to the bulk soil.

### The soil properties influencing the keystone taxa

**Figure 7:**
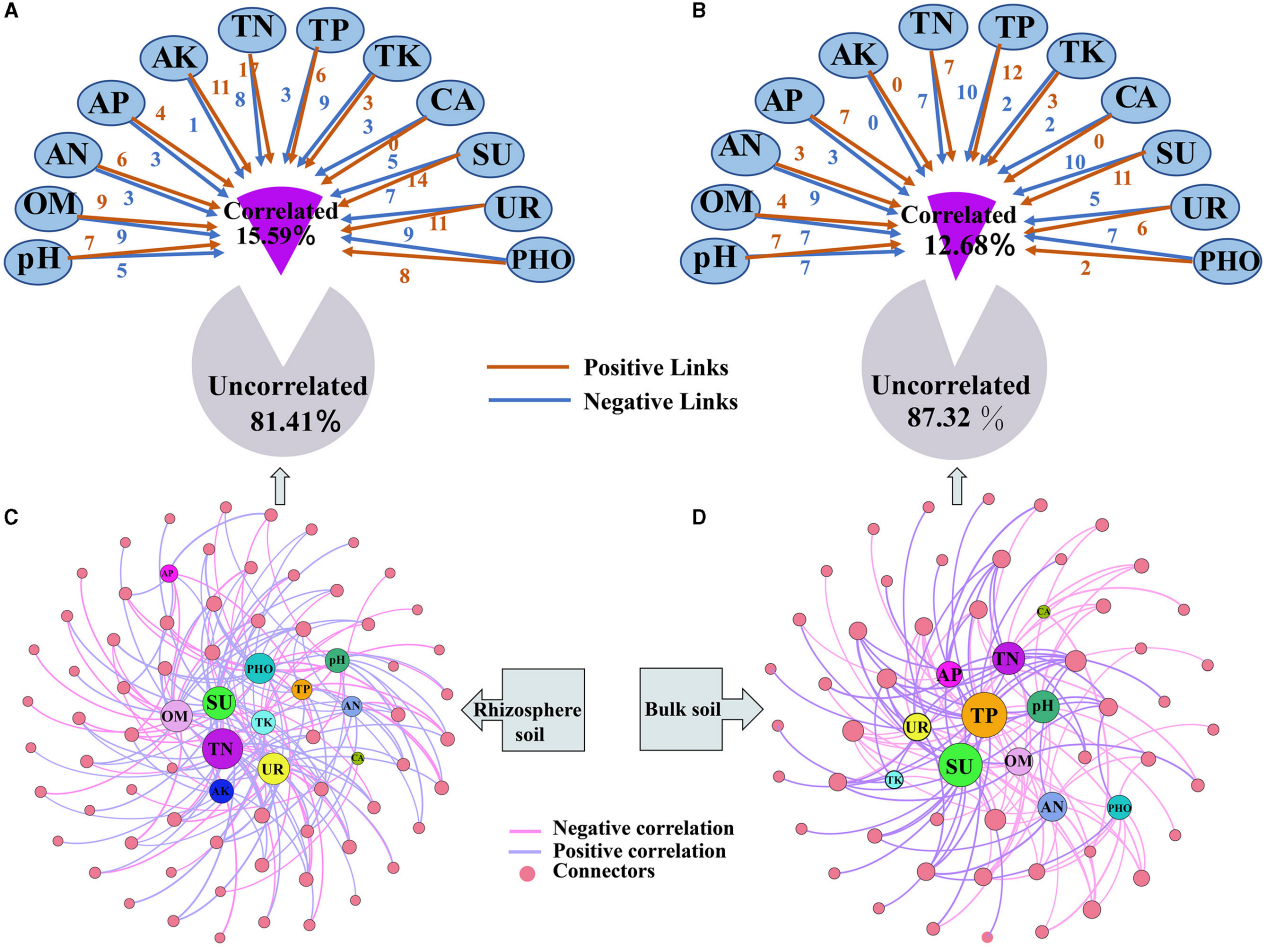
Influence of soil properties on keystone taxa in both rhizosphere and bulk soils. **(A, B)** represent the impact of soil properties on network connectors; **(C, D)** represent the correlation network between network connectors and soil properties.

A new network that examines the relationship between keystone taxa in relative abundances and soil properties was constructed (Figure 7 and Supplementary Table S13). Soil properties were found not to significantly correlate with most keystone taxa in the rhizosphere (81.41%) and bulk soils (87.32%). However, when significant correlations were present, the strength of the correlations in the rhizosphere increased (Figures 7A, B). Upon examining the correlations, it was observed that the keystone taxa showed a higher ratio of positive correlations with soil properties compared with negative correlations. Positive correlations were more prevalent in the rhizosphere (59.63%) than in the bulk soil (47.33%) (Supplementary Table S13). Among the soil properties, the relationship correlation between TN, SU, UR, OM, PHO, and pH with keystone taxa was higher than those of other soil properties in the rhizosphere (Figure 7C). On the other hand, the correlation between TP, SU, TN, pH, and OM with keystone taxa was higher than that of other soil properties in the bulk soil (Figure 7D).

## Discussion

### The rhizosphere soil has higher nutrient and enzyme activity

Based on the research findings, it was observed that the rhizosphere of Chinese cabbage had a higher nutrient content and enzyme activity compared with the bulk soils in the *Karst* region. The pH level showed the opposite pattern, consistent with a previous study (Kuzyakov and Razavi, 2019). Enzyme activity typically decreases with distance from the root surface, primarily releasing organic C (Kuzyakov and Razavi, 2019). Earlier studies have shown that the ratio of sedimentary carbon in the rhizosphere, originating from roots and sediments, accounts for ~54–63% of the total carbon in grains (Hirte et al., 2018). The unstable nature of this carbon enhances the activity of rhizosphere microbes compared with bulk soils, leading to elevated enzyme activity in the rhizosphere (Jat et al., 2021). Additionally, different enzymes are directly and significantly influenced by soil properties, such as nutrient contents, available C, biomass, and microbial activity, resulting in higher enzyme activities in the rhizosphere soil compared with the bulk soil (Kuzyakov and Razavi, 2019). Moreover, the rhizosphere releases acidic substances, which reduce the pH levels in the soil surrounding the roots (Mendes et al., 2014). Conversely, Chen et al. (2019) found that an increase in organic acid content was accompanied by a corresponding increase in organic carbon. Similar results were reported for tea and maize plants in the rhizosphere soils by Kong et al. (2020) and Yan et al. (2018). Organic matter undergoes a faster transformation, resulting in higher accumulation in the rhizosphere soil (Sokol et al., 2022). As organic matter mineralizes, it releases nitrogen, phosphorus, and potassium, resulting in increased concentrations in both the rhizosphere and bulk soils. Additionally, carbon and nitrogen tend to accumulate together in the soil, where an increase in carbon content promotes nitrogen accumulation (Tang et al., 2023). The research established a significant positive association between organic matter and essential nutrients (AN and TN, AP and TP, and AK and TK), providing insights into why the rhizosphere soil typically displays higher nutrient levels and greater enzyme activity compared with the bulk soil.

### Composition and diversity of bacterial and fungal communities

Recently conducted research has reported similar results to those found in this study, confirming that both the rhizosphere and bulk soils of Chinese cabbage in the *Karst* region exhibit lower bacterial and fungal alpha diversity and OTUs (Zhang et al., 2018b). These findings add further evidence to the notion that the plant's preference for attracting rhizosphere-colonizing microorganisms contributes to the reduced alpha diversity observed within the rhizosphere. This leads to a shift in species richness and decreased species homogeneity in the rhizosphere soil (Ling et al., 2022). As expected, bacteria are found more commonly in different environments than fungi. This study confirms this trend, with the rhizosphere soil being dominated by several bacterial phyla, including Proteobacteria (37.59%), Acidobacteriota (14.74%), Actinobacteriota (10.73%), and Bacteroidota (9.87%). In contrast, bulk soil is dominated by *Proteobacteria* (23.60%), *Acidobacteriota* (20.60%), *Chloroflexi* (16.03%), and *Actinobacteriota* (13.00%). Notably, the relative abundance of *Proteobacteria* in the rhizosphere soil is nearly double the amount in the bulk soil. *Proteobacteria* and *Bacteroidota* thrive in carbon-rich environments and are more abundant in the rhizosphere due to their high metabolic activity, rapid growth, and reproduction (Ling et al., 2022). Additionally, many anamorphic bacteria, which belong to the gram-negative phylum, play a crucial role in nitrogen fixation, leading to increased accumulation of nitrogen in the soil (Ladha and Reddy, 2003). The higher relative abundance of *Proteobacteria* and *Bacteroidota* in the rhizosphere soil suggests greater metabolic activity and higher nutrient availability compared with the bulk soil. Regarding fungi, the research showed that the dominant phyla were Olpidiomycota, Ascomycota, Mortierellomycota, and Basidiomycota in the rhizosphere and bulk soils, with varying relative abundance. *Olpidiomycota* was found to be dominant in the rhizosphere soils, whereas *Ascomycota* was dominant in the bulk soils. Fungi play a crucial role in plant and animal remains decomposition (Xiong et al., 2021), and an increase in the relative abundance of fungal *Ascomycetes* has been linked to enhanced plant debris decomposition (Wang et al., 2020). *Olpidiomycota*, represented by *Olpidium*, includes pathogens (Lay et al., 2018). Interestingly, LEfSe analysis revealed higher levels of *Olpidium, Olpidiaceae*, and *Olpidiomycetes* in the Chinese cabbage rhizosphere compared with adjacent bulk soils. Functional analysis further showed higher fungal pathotrophs in the rhizosphere soil, while fungal saprotrophs were considerably more prevalent in the bulk soil, nearly 10 times higher in relative abundance (Supplementary Table S9). Saprophytic fungi play an important role in breaking down plant debris, promoting decomposition of organic matter, and cycling of nutrients (Egidi et al., 2019). On the other hand, pathogenic fungi can cause damage to both plants and the soil (Chen et al., 2020). The findings suggest that the bulk soil is the primary site for plant residue decomposition, while the rhizosphere soil may harbor a higher concentration of potential pathogens. Several reasons may explain this phenomenon: First, a significant proportion of plant pathogens thrive in a saprophytic manner in the roots and thus derive energy from the rhizosphere (Larsen et al., 2015); Second, certain pathogens are more likely to colonize the rhizosphere based on the research studies by Hannula et al. (2021) and Ma et al. (2020); Third, diverse sediments found in the rhizosphere, such as carboxylic acids, sugars, amino acids, and polymeric carbohydrates, create a favorable environment for the proliferation and survival of pathogens.

### Rhizosphere soil has a simpler co-occurrence network

The co-occurrence network in the rhizosphere soil of Chinese cabbage displayed a weaker structure compared with that in the bulk soil. The bacterial–fungal network in the rhizosphere soil exhibited a higher number of positive correlations and lower complexity, as expected. This aligns with the notion that the microbial community in the rhizosphere soil is a subset of the bulk soil, a common trait among various plant species (Ling et al., 2022). Previous research studies on soybean farming in different regions of China also reported a smaller and less intricate rhizosphere network compared with the bulk soil (Zhang et al., 2021). Furthermore, Ling et al. (2022) found that the rhizosphere network displayed less robustness and stability, consistent with these results. This could be attributed to increased rhizosphere resources, leading to reduced overlap and interaction among rhizosphere niches (Zhang et al., 2018a). As a result, microbial interactions in the rhizosphere decreased, enabling more microbiomes to adopt a free lifestyle leading to a simpler and weaker stable co-occurrence pattern. Furthermore, as the alpha diversity of bacteria and fungi decreased from the bulk soil to the rhizosphere soil, the complexity of the rhizosphere network also reduced (Fan et al., 2018). Keystone taxa play an irreplaceable role in shaping the co-occurrence network structure stability (Shi et al., 2016). For instance, Proteobacteria, a bacterial phylum, can rapidly build connections and utilize a majority of the carbon substrates released by roots (Philippot et al., 2013). Agaricomycetes and Dothideomycetes fungi possess connector species that enhance nutrient absorption, offer protection against pathogenic bacteria, and maintain harmonious metabolic relationships with other species (Gueidan et al., 2009). These keystone taxa exhibit a versatile, generalist metabolism that helps maintain a balanced internal environment (Fan et al., 2018). The study results indicate that the bulk soil displays greater complexity in bacterial, fungal, and bacterial–fungal networks, compared with the rhizosphere soil. Moreover, the impact of the rhizosphere soil was discovered to be more significant on fungi than bacteria. Notably, a higher number of OTUs linked to Burkholderiales and Rhizobiales are found in the rhizosphere soil, which constitutes the core root microbiome of terrestrial plants (Ji et al., 2023). Rhizobiales have been identified as endophytes in different plants and have been shown to contribute to the growth of crops such as rice and wheat (Yanni et al., 2016; Wu et al., 2018). Moreover, the keystone taxa in the bulk soil exhibited a weak correlation with soil properties. Rhizosphere soil microbial communities are more vulnerable to disturbances under changing environmental conditions compared with bulk soil. The simpler structure of the network in the rhizosphere soil makes it challenging for the ecosystem to recover when exposed to external disruptions.

## Conclusion

Through the study of the microbial communities in the rhizosphere and bulk soils of Chinese cabbage in the *Karst* regions, significant differences were observed in their composition. Specifically, the microbiome in the rhizosphere was a subset of the bulk soil, exhibiting lower alpha diversity than the latter. Moreover, the co-occurrence network in the rhizosphere soil displayed lower complexity, and keystone taxa showed a stronger correlation with soil properties compared with the bulk soil. The functional analysis revealed a higher proportion of pathogens in the relative abundance among the bacterial community in the bulk soil, while the opposite tendency was discovered for fungi in the rhizosphere soil. Despite the reduced complexity of the rhizosphere network, a higher prevalence of beneficial bacterial species that promote the growth of Chinese cabbage was observed.

## Data availability statement

The datasets presented in this study can be found in online repositories. The names of the repository/repositories and accession number(s) can be found in the article/Supplementary material.

## Author contributions

XW and TF conceived the ideas and designed the methodology. GH, ZZ, MY, and FL completed validation. XW and TH wrote the manuscript. All authors contributed to the article and approved the submitted version.
