# Supplementary material for: Characteristics of rhizosphere and bulk soil microbial community of Chinese cabbage (Brassica campestris) grown in Karst area
Source: Front Microbiol. 2023 Sep 15;14:1241436. doi: 10.3389/fmicb.2023.1241436 (PMC10542900; doi:10.3389/fmicb.2023.1241436)
Supplement: Supplementary file 1 [file Data_Sheet_1.doc]

Characteristics of rhizosphere and bulk soil microbial community of Chinese cabbage (*Brassica campestris*) grown in *Karst* area

Xiaoliao Wei^1^, Tianling Fu^2^, Guandi He^1^, Zhuoyan Zhong^1^, Mingfang Yang^1^, Fei Lou^1^, Tengbing He^1,2*^

^1^ College of Agriculture, Guizhou University, Guiyang, 550025, PR China

^2^ Institute of New Rural Development, Engineering Key Laboratory for Pollution Control and Resource Reuse Technology of Mountain Livestock Breeding, Guizhou University, Guiyang, 550025, PR China

**Supplementary methods**

**16S rRNA gene and ITS rRNA gene amplification:**

For bacterial 16S rRNA gene amplification, the V3-V4 region was targeted utilizing the 338F (5′-ACTCCTACGGGAGGCAGCAG-3′) and 806R (5′-GGACTACHVGGGTWTCTAAT-3′) primers (Liu et al., 2016); the PCR reactions were performed utilizing TransGen AP221-02: TransStart Fastpfu DNA Polymerase (ABI, USA) in a 20µl reaction system. The reaction mixture contained 4ul 5×FastPfu Buffer, 2ul 2.5 mM dNTPs, 0.8 µl Forward Primer(5 µM), 0.8 µl Reverse Primer(5 µM), 0.4 µl FastPfu Polymerase，0.2 µl BSA, 10 ng Template DNA, make up ddH_2_O to 20 µl; PCR reaction parameters were a. 1× (3 minutes at 95 ℃), b. 27 × (30 seconds at 95 ℃; 30 seconds at 55 ℃; 45 seconds at 72 ℃), c. 10 minutes at 72 ℃, and 10 ℃ until stopped by the user.

For ITS rRNA gene amplification, the ITS1 region was targeted using ITS1F (5′-CTTGGTCATTTAGAGGAAGTAA-3′) and ITS2R (5′-GCTGCGTTCTTCATCGATGC-3′) primers (Gardes and Bruns, 1993; White et al., 1990). The PCR reactions were performed using TaKaRa rTaq DNA Polymerase, in a 20µl reaction system. The PCR mixure contained 2 µl 10× Buffer, 2 µl 2.5 mM dNTPs, 0.8µl Forward Primer(5 µM), 0.8µl Reverse Primer (5 µM), 0.2 µl rTaq Polymerase, 0.2 µl BSA, 10 ng Template DNA, make up ddH_2_O to 20 µl; PCR reaction parameters were a. 1× (3 minutes at 95 ℃), b. 35 × (30 seconds at 95℃; 30 seconds at 55 ℃; 45 seconds at 72 ℃), c. 10 minutes at 72 ℃，and 10 ℃ until stopped by the user.

**Data processing:**

The raw fastq files were processed using QIIME to demultiplex and quality-filter them based on specific condictions (Caporaso et al., 2010): (i) filter the bases with mass value below 20 at the end of the reads, set a window of 50bp, if the average mass value within the window is below 20, truncate the back-end bases starting from the window, filter the reads with a mass value below 50bp after quality control, and remove the reads containing N bases; (ii) based on the overlap relationship between PE reads, pairs of reads are spliced (merged) into a sequence with a minimum overlap length of 10bp; (iii) The maximum mismatch ratio allowed in the overlap region of a spliced sequence is 0.2, screening for non-conforming sequences; (iv) the samples were differentiated according to the barcode and primers at the beginning and end of the sequence and the sequence orientation was adjusted, with an allowable number of mismatches of 0 for the barcode and a maximum of 2 for the primer mismatches

Caporaso, J. G., Kuczynski, J., Stombaugh, J., Bittinger, K., Bushman, F. D., Costello, E. K., Fierer, N., Peña, A. G., Goodrich, J. K., Gordon, J. I., Huttley, G. A., Kelley, S. T., Knights, D., Koenig, J. E., Ley, R. E., Lozupone, C. A., McDonald, D., Muegge, B. D., Pirrung, M., Reeder, J., Sevinsky, J. R., Turnbaugh, P. J., Walters, W. A., Widmann, J., Yatsunenko, T., Zaneveld, J., & Knight, R. (2010). QIIME allows analysis of high-throughput community sequencing data. Nature Methods, 7, 335-336. https://doi.org/10.1038/nmeth.f.303

Gardes, M., & Bruns, T. D. (1993). ITS primers with enhanced specificity for basidiomycetes--application to the identification of mycorrhizae and rusts. Mol Ecol, 2, 113-118. https://doi.org/10.1111/j.1365-294x.1993.tb00005.x

Liu, C., Zhao, D., Ma, W., Guo, Y., Wang, A., Wang, Q., & Lee, D. J. (2016). Denitrifying sulfide removal process on high-salinity wastewaters in the presence of Halomonas sp. Applied microbiology and biotechnolog, 100, 1421-1426. https://doi.org/10.1007/s00253-015-7039-6

White T, Bruns T, Lee S, Taylor J.1990Amplification and direct sequencing of fungal ribosomal RNA genes for phylogeneticsIn: Innis N, Gelfand D, Sninsky J, White T (eds)PCR: Protocols and Applications—A Laboratory Manual Academic Press: New York; 315–322.


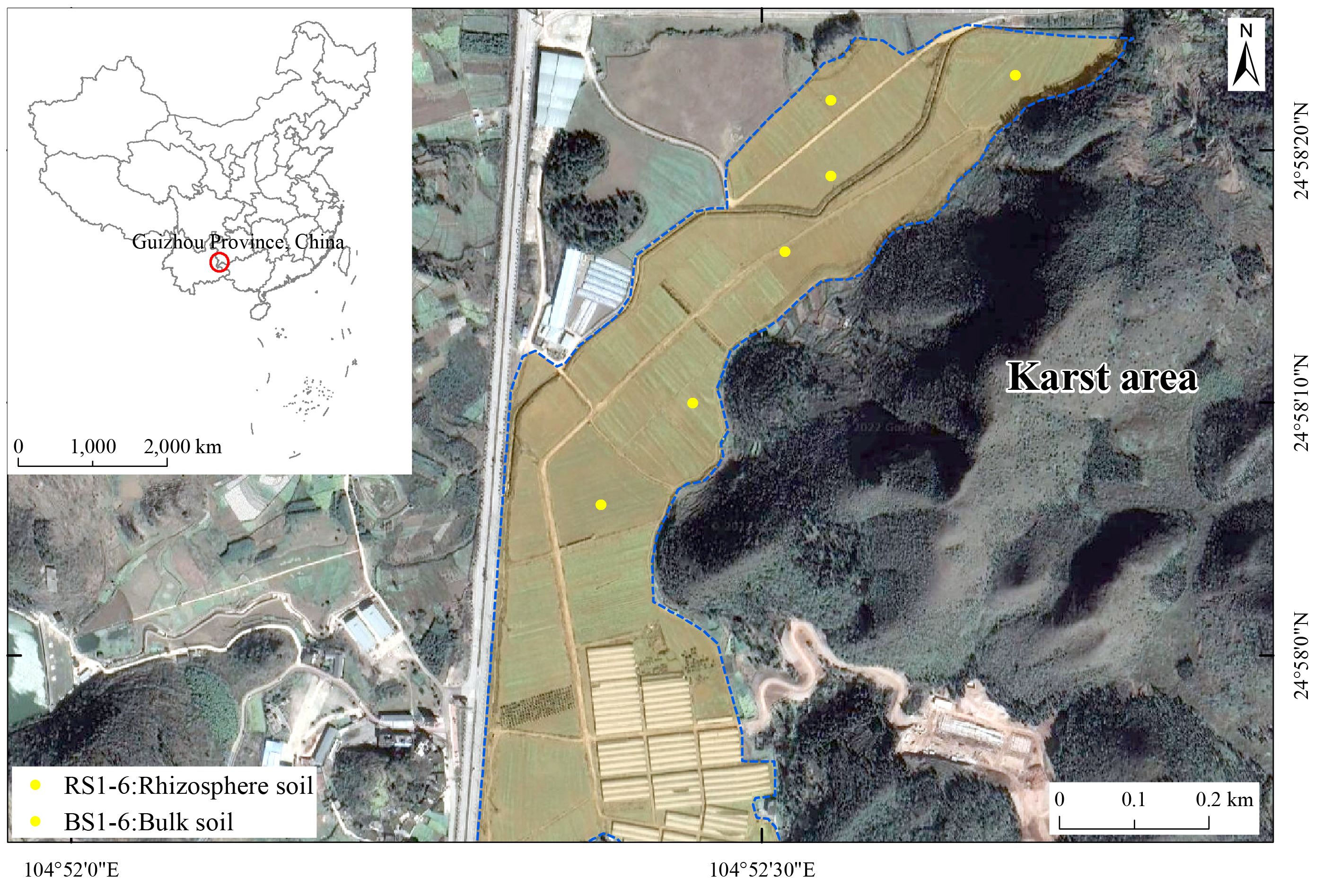
**Figure S1** Satellite imagery of sampling point layout


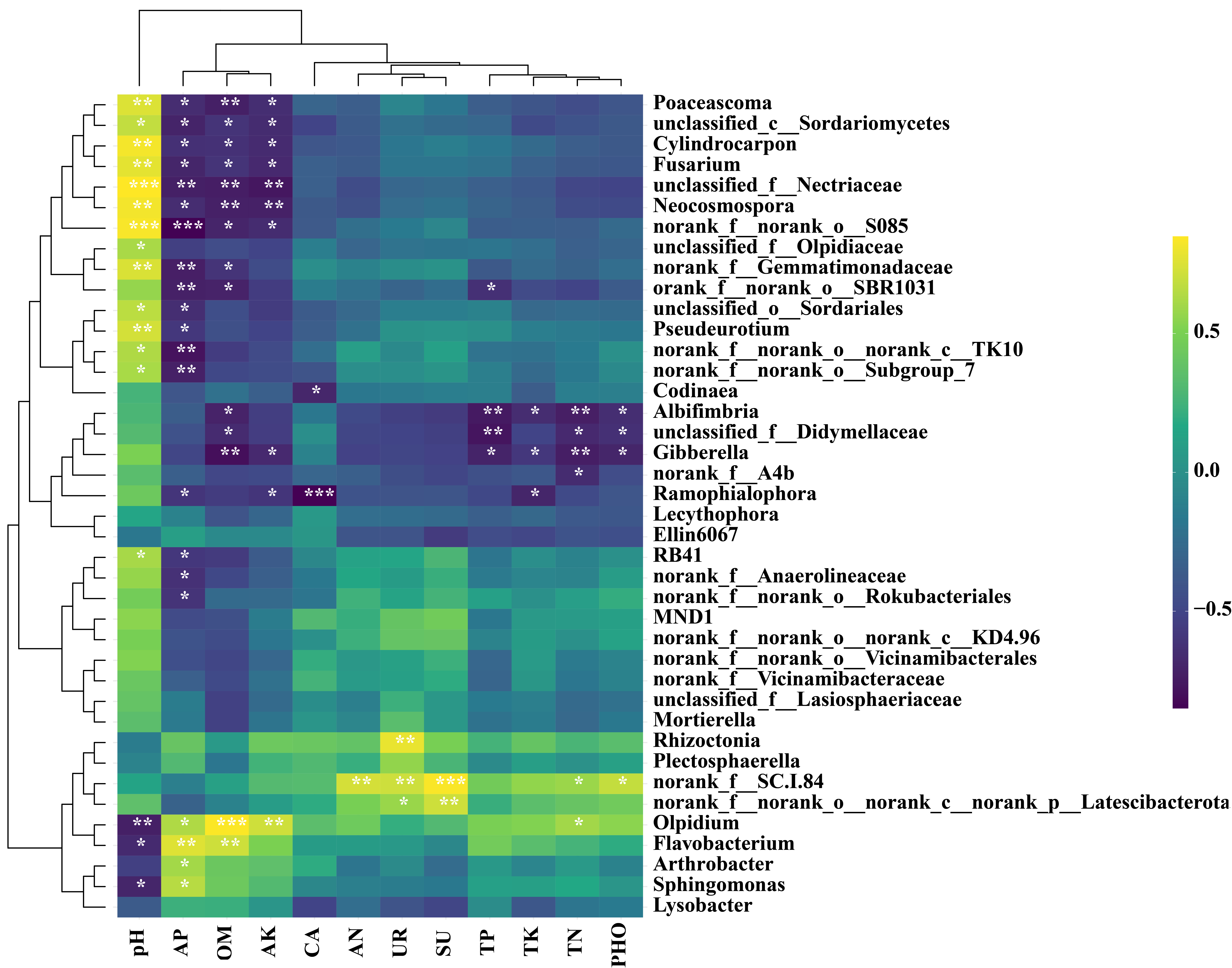


**Figure S2** Pearson correlation of microbial genera and soil environmental factors. Note: "***" shows at p ≤ 0.001, "**" at shows 0.001 < p≤ 0.01, "*" shows at 0.01 < p≤ 0.05.


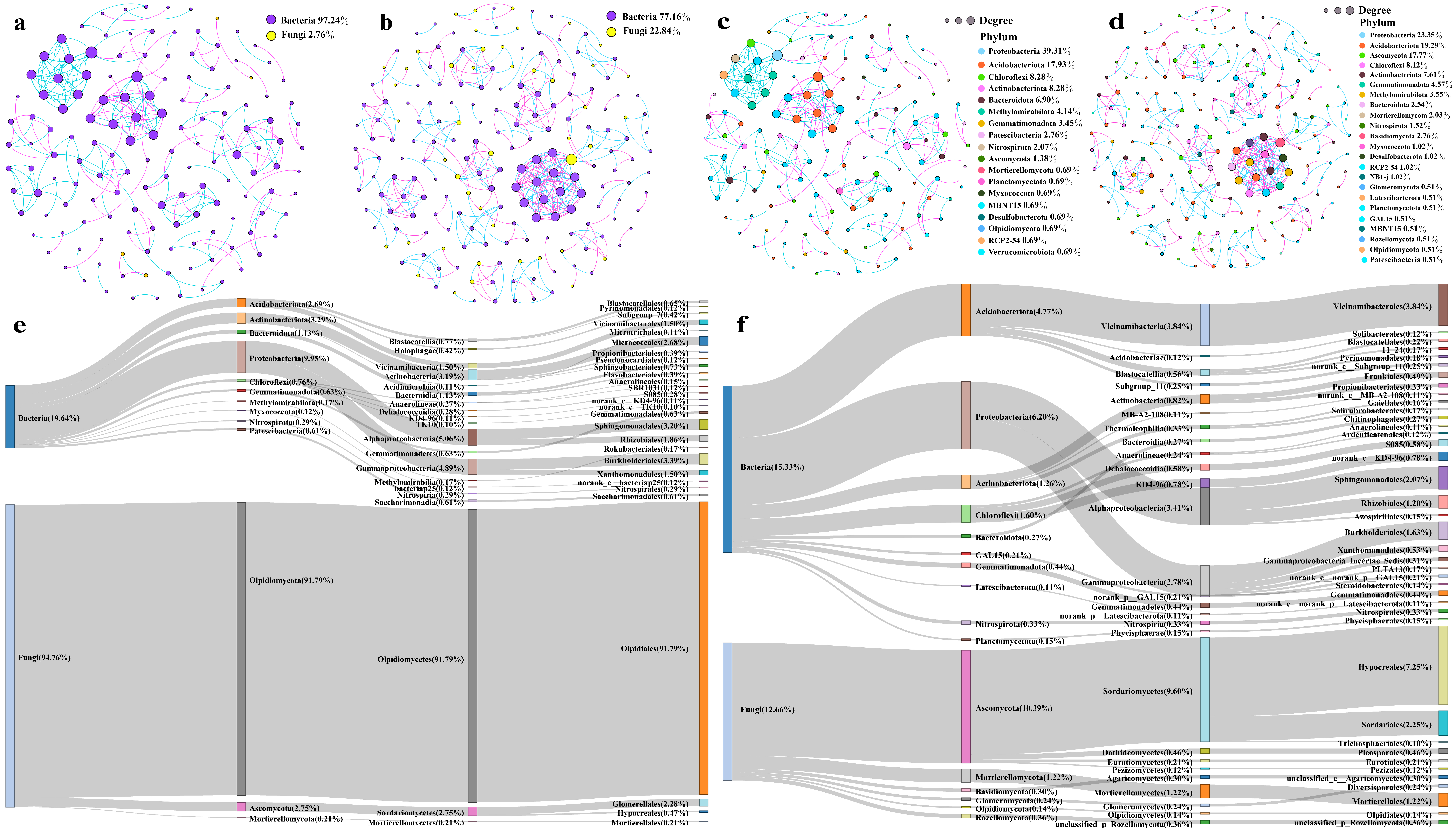


Figure S3 Co-occurrence network of bacteria-fungi in the Chinese cabbage in the rhizosphere and bulk soils. Bacterial and fungal proportion in the rhizosphere (a) and bulk (b) soils bacterial-fungal inter-kingdom co-occurrence network of Chinese cabbage; distribution of nodes in the rhizosphere (c) and bulk (d) soils bacterial-fungal inter-kingdom co-occurrence network of Chinese cabbage; distribution of keystone taxa (from phylum to order) in the Chinese cabbage rhizosphere soil bacterial-fungal inter-kingdom co-occurrence network (e); distribution of keystone taxa (from phylum to order) in the Chinese cabbage bulk soil bacterial-fungal inter-kingdom co-occurrence network (f). Nodes are colored according to bacterial and fungal phyla, and edges represent correlations between nodes; purple indicates positive correlation while blue indicates negative correlation.


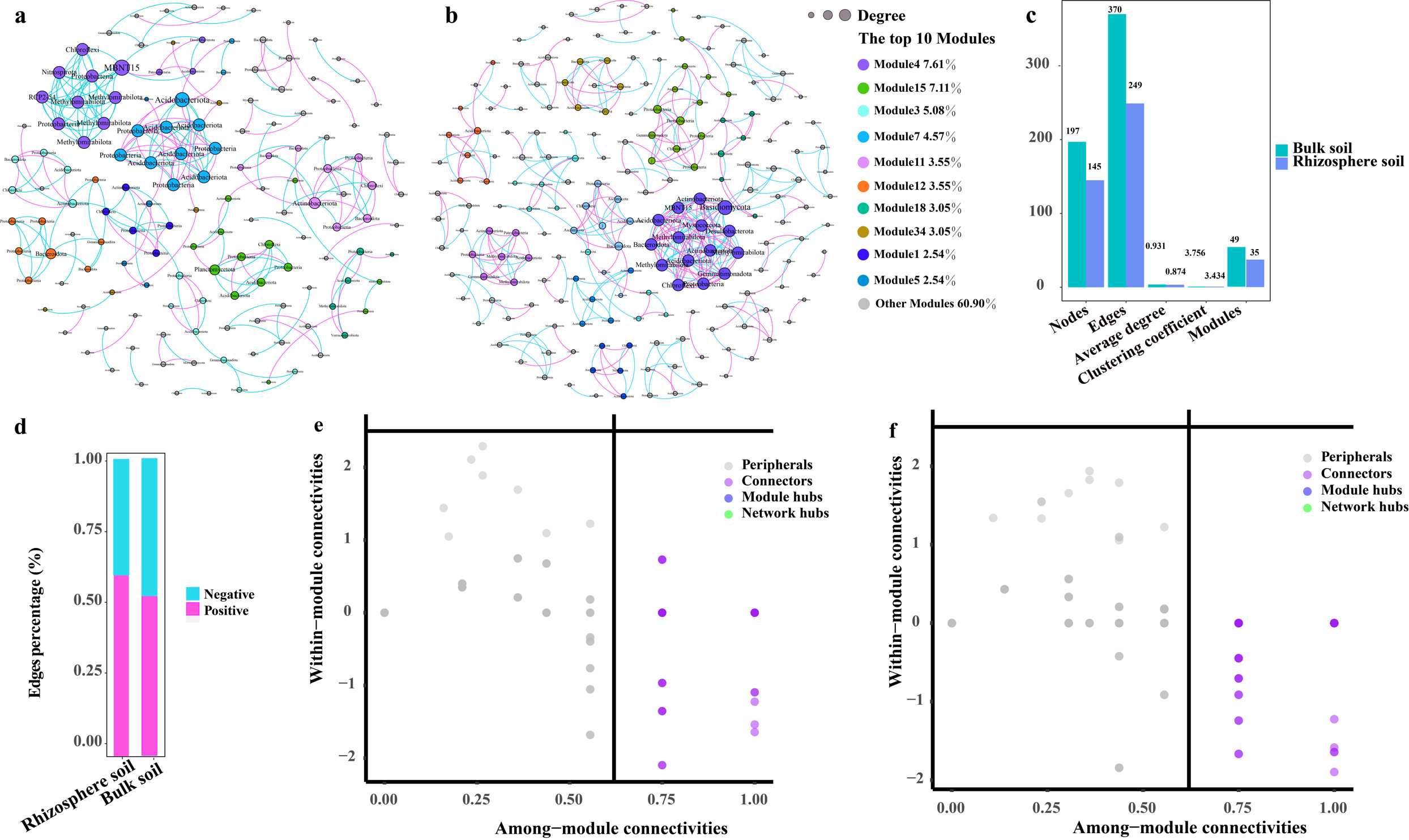
**Figure S4** Topological properties and key taxa of bacterial-fungal co-occurrence networks in rhizosphere and bulk soils. Nodes are colored according to the microbial phyla, the same color represents the same module. Edges represent correlations between nodes; purple represents positive correlation and blue represents negative correlation.
